# 10-Year Fracture Risk Assessment with Novel Adjustment (FRAXplus): Type 2 Diabetic Sample-Focused Analysis

**DOI:** 10.3390/diagnostics15151899

**Published:** 2025-07-29

**Authors:** Oana-Claudia Sima, Ana Valea, Nina Ionovici, Mihai Costachescu, Alexandru-Florin Florescu, Mihai-Lucian Ciobica, Mara Carsote

**Affiliations:** 1 PhD Doctoral School of “Carol Davila” University of Medicine and Pharmacy, 010825 Bucharest, Romania; oana-claudia.sima@drd.umfcd.ro; 2 Department of Endocrinology, “Iuliu Hatieganu” University of Medicine and Pharmacy, 400012 Cluj-Napoca, Romania; 3 Department of Endocrinology, County Emergency Clinical Hospital, 400347 Cluj-Napoca, Romania; 4 Occupational Medicine Department, University of Medicine and Pharmacy of Craiova, 200349 Craiova, Romania; nina.ionovici@umfcv.ro; 5 Department of Radiology and Medical Imaging, “Dr. Carol Davila” Central Emergency University Military Hospital, 010825 Bucharest, Romania; 6 Department of Endocrinology, “Grigore T. Popa” University of Medicine and Pharmacy, 700111 Iasi, Romania; alexandru-florin.florescu@umfiasi.ro; 7 Department of Internal Medicine and Gastroenterology, “Carol Davila” University of Medicine and Pharmacy, 020021 Bucharest, Romania; lucian.ciobica@umfcd.ro; 8 Department of Internal Medicine I and Rheumatology, “Dr. Carol Davila” Central Military University Emergency Hospital, 010825 Bucharest, Romania; 9 Department of Endocrinology, “Carol Davila” University of Medicine and Pharmacy, 020021 Bucharest, Romania; carsote_m@hotmail.com

**Keywords:** diabetes, bone, fracture, FRAX, bone mineral density, menopause, mineral metabolism, DXA, fracture risk, 10-year probability of fracture risk, FRAXplus

## Abstract

**Background**: Type 2 diabetes (T2D) has been placed among the risk factors for fragility (osteoporotic) fractures, particularly in menopausal women amid modern clinical practice. **Objective**: We aimed to analyze the bone status in terms of mineral metabolism assays, blood bone turnover markers (BTM), and bone mineral density (DXA-BMD), respectively, to assess the 10-year fracture probability of major osteoporotic fractures (MOF) and hip fracture (HF) upon using conventional FRAX without/with femoral neck BMD (MOF-FN/HF-FN and MOF+FN/HF+FN) and the novel model (FRAXplus) with adjustments for T2D (MOF+T2D/HF+T2D) and lumbar spine BMD (MOF+LS/HF+LS). **Methods**: This retrospective, cross-sectional, pilot study, from January 2023 until January 2024, in menopausal women (aged: 50–80 years) with/without T2D (group DM/nonDM). Inclusion criteria (group DM): prior T2D under diet ± oral medication or novel T2D (OGTT diagnostic). Exclusion criteria: previous anti-osteoporotic medication, prediabetes, insulin therapy, non-T2D. **Results**: The cohort (N = 136; mean age: 61.36 ± 8.2y) included T2D (22.06%). Groups DM vs. non-DM were age- and years since menopause (YSM)-matched; they had a similar osteoporosis rate (16.67% vs. 23.58%) and fracture prevalence (6.66% vs. 9.43%). In T2D, body mass index (BMI) was higher (31.80 ± 5.31 vs. 26.54 ± 4.87 kg/m^2^; *p* < 0.001), while osteocalcin and CrossLaps were lower (18.09 ± 8.35 vs. 25.62 ± 12.78 ng/mL, *p* = 0.002; 0.39 ± 0.18 vs. 0.48 ± 0.22 ng/mL, *p* = 0.048), as well as 25-hydroxyvitamin D (16.96 ± 6.76 vs. 21.29 ± 9.84, *p* = 0.013). FN-BMD and TH-BMD were increased in T2D (*p* = 0.007, *p* = 0.002). MOF+LS/HF+LS were statistically significant lower than MOF-FN/HF-FN, respectively, MOF+FN/HF+FN (N = 136). In T2D: MOF+T2D was higher (*p* < 0.05) than MOF-FN, respectively, MOF+FN [median(IQR) of 3.7(2.5, 5.6) vs. 3.4(2.1, 5.8), respectively, 3.1(2.3, 4.39)], but MOF+LS was lower [2.75(1.9, 3.25)]. HF+T2D was higher (*p* < 0.05) than HF-FN, respectively, HF+FN [0.8(0.2, 2.4) vs. 0.5(0.2, 1.5), respectively, 0.35(0.13, 0.8)] but HF+LS was lower [0.2(0.1, 0.45)]. **Conclusion**: Type 2 diabetic menopausal women when compared to age- and YSM-match controls had a lower 25OHD and BTM (osteocalcin, CrossLaps), increased TH-BMD and FN-BMD (with loss of significance upon BMI adjustment). When applying novel FRAX model, LS-BMD adjustment showed lower MOF and HF as estimated by the conventional FRAX (in either subgroup or entire cohort) or as found by T2D adjustment using FRAXplus (in diabetic subgroup). To date, all four types of 10-year fracture probabilities displayed a strong correlation, but taking into consideration the presence of T2D, statistically significant higher risks than calculated by the traditional FRAX were found, hence, the current model might underestimate the condition-related fracture risk. Addressing the practical aspects of fracture risk assessment in diabetic menopausal women might improve the bone health and further offers a prompt tailored strategy to reduce the fracture risk, thus, reducing the overall disease burden.

## 1. Introduction

During the last decade, type 2 diabetes mellitus has been placed among the risk factors for fragility (osteoporotic) fractures, particularly in menopausal women [1,2,3]. Central Dual-Energy X-Ray Absorptiometry (DXA) might not always capture the true essence of this elevated fracture risk since bone mineral density (BMD) is correlated with body mass index (BMI), which might be high in many of these patients with increased cardio-metabolic risk, including obesity [4,5,6]. Other practical aspects of bone heath evaluation may include a reduced level of bone turnover markers (BTMs), decreased bone microarchitecture (as shown by lumbar DXA-derivate trabecular bone score), and an elevated risk of fall due to glycaemia and blood pressure variations, neuropathy, sarcopenia, as well as multiple eye complications in diabetic subjects [7,8,9].

10-year probability of fracture is provided by various risk calculators. For instance, FRAX (Fracture Risk Assessment Tool) [10] offers an estimation of major osteoporotic fractures (MOF) and hip fracture (HF) in individuals 40 years or older, based on pivotal inputs that represent well-established fracture risks such as age, prevalent fragility fractures, daily habits (e.g., smoking or alcohol drinking in certain amounts), glucocorticoid exposure, etc. [10,11,12]. Notably, the results are calculated with or without introducing femoral neck BMD. Overall, a potential gap in subjects with asymmetrical deterioration of lumbar BMD versus other central (non-lumbar) sites might be found (meaning the lumbar site might be affected to a lesser degree than total hip or femoral neck or the other way around). Moreover, while the algorithm takes into consideration numerous causes of secondary bone loss [e.g., osteogenesis imperfecta in adults; type 1 (insulin-dependent) diabetes; untreated hypogonadism and long-standing hyperthyroidism; chronic liver, kidney failure, etc.], type 2 diabetes (and the potential impact of its duration) is missing from the conventional model [13,14,15]. FRAXplus is currently under beta testing for novel adjustments of the previous model (e.g., with inputs such as lumbar BMD, type 2 diabetes, number of falls in the previous year, presence of a recent fragility fracture, enhanced exposure to glucocorticoid medication, etc.), and is a work in progress that is intended to expand previous estimations [16].

### Objective

We aimed to analyze the bone status on menopausal type 2 diabetic versus non-diabetic subjects in terms of mineral metabolism assays, BTMs, and central DXA, as well as to assessing the 10-year fracture probability of MOF and HF upon using the conventional FRAX model and the novel algorithm (FRAXplus) with adjustments regarding type 2 diabetes and lumbar BMD.

## 2. Material and Methods

**Study design**: was retrospective, cross-sectional, pilot study, from January 2023 until January 2024.

**Study population**: Menopausal women with/without type 2 diabetes were analyzed according to the inclusion/exclusion criteria after having at least one hospitalization.

**Inclusion criteria** were: females who were confirmed with menopausal status, and an age between 50 and 80 years; the presence of central DXA evaluation. They signed the written consent (as inpatient).

**Exclusion criteria** were: prior diagnosis of osteoporosis, prior or current exposure to anti-osteoporotic medication (e.g., oral or intravenous bisphosphonates, denosumab, teriparatide, romosozumab, calcitonin); end-stage kidney disease, active malignancies or endocrine tumors (including primary hyperparathyroidism, acromegaly and Cushing’s syndrome); prior or current hormone replacement therapy (for menopause) or current glucocorticoids, insulin therapy, thiazolidinedione or glucagon-like peptide-1 receptor agonists; lack of data/inconsistent records with respect to the history of diabetes or glucose profile assessments; other forms of diabetes (non-type 2 diabetes); acute diabetic complications of any category during hospitalization; acute infections; central DXA (lumbar, femoral neck and total hip) assessment was non-interpretable (due to artefacts or overlapping images such as kidney stones, scoliosis, hip prosthesis, etc.); pre-diabetes. Of note, all the mentioned drugs were excluded due to clear impact on bone status that might produce a bias in assessing diabetic status (e.g., estrogens, glucocorticoids, insulin) or their effects on the fracture risk have not been clearly established yet (e.g., glucagon-like peptide-1 receptor agonists).

**Studied sub-groups** designation: The entire cohort was assigned as “sub-group DM” [patients with type 2 diabetes that was confirmed before the actual hospitalization (the disease being under diet and/or oral anti-diabetic drugs)] or newly detected type 2 diabetes based on 75-g oral glucose tolerance test (OGTT) that revealed a 2-h glycaemia of at least 200 mg/dL [17] versus sub-group “nonDM” (patients who were ruled out as diabetics and they were considered control group).

**Study protocol**: The patients who were prior known with type 2 diabetes were not re-tested via OGTT, while all the other subjects underwent an OGTT. Individuals confirmed as diabetic (using OGTT) were included in sub-group DM. Subjects who had a normal OGTT were included in control (nonDM) sub-group, while those with prediabetes (impaired glucose tolerance or impaired fasting glycaemia [17]) were excluded. Also, the individuals who were not previously confirmed as type 2 diabetic, but were not able or did not undergo OGGT were ruled out.

**Figure 1:**
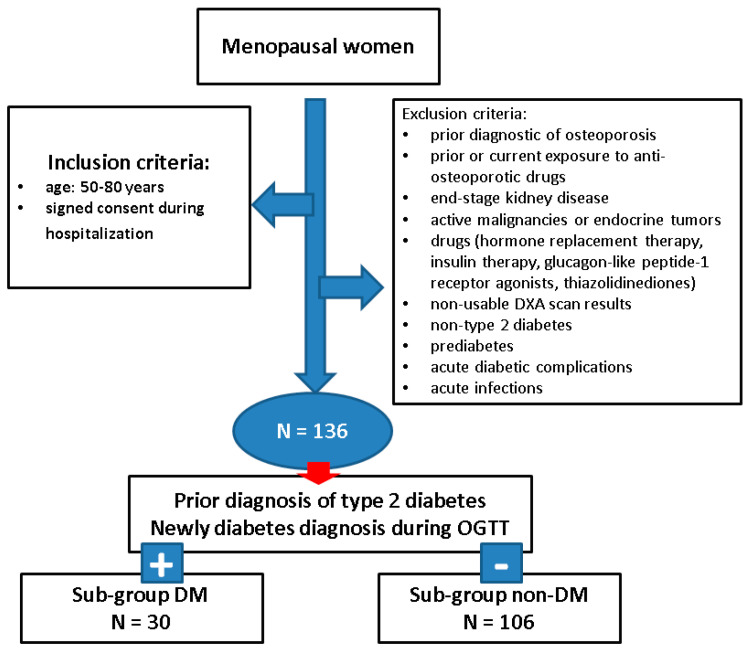
Flowchart diagram of the study.

**Assessments during study analysis**: The retrospective data collection included: age (years), years since menopause, BMI (kg/m^2^), the diagnosis of type 2 diabetes (prior diagnosis or current confirmation using OGTT as shown above), and dyslipidemia (any type; registered as “yes” or “no”); prevalent osteoporotic (fragility) fractures (according to the previous medical records of the patients and/or thoracic-lumbar spine X-ray that was performed on admission or within the previous 6 months) (Figure 1).

The blood mineral metabolism evaluation [total and ionic serum calcium, phosphorus, magnesium, parathormone (PTH), 25-hydroxyvitamin D] and serum BTM profile [osteocalcin, P1NP, alkaline phosphatase (formation markers), and CrossLaps (resorption marker)] was registered, as well as A1c glycated hemoglobin (%). Central DXA scans (GE Lunar Prodigy device) provided lumbar, femoral neck and total hip BMD/T-score for each patient [18,19]. Screening X-Rays of the spine and DXA scans were re-analyzed by a trained radiologist (M.C. (Mihai Costachescu)) within the current study in addition to the baseline (initial) evaluation from each hospital.

Osteoporotic fractures were appreciated as fractures that spontaneously occurred or, alternatively, fractures that occurred during a fall from a height equal to the patient’s height. Moreover, completely asymptomatic vertebral fractures that were detected at screening X-Ray of the thoracic-lumbar spine region were also included in this category. The study data collection concerning the fractures was done as “yes” or “no”.

**Figure 2:**
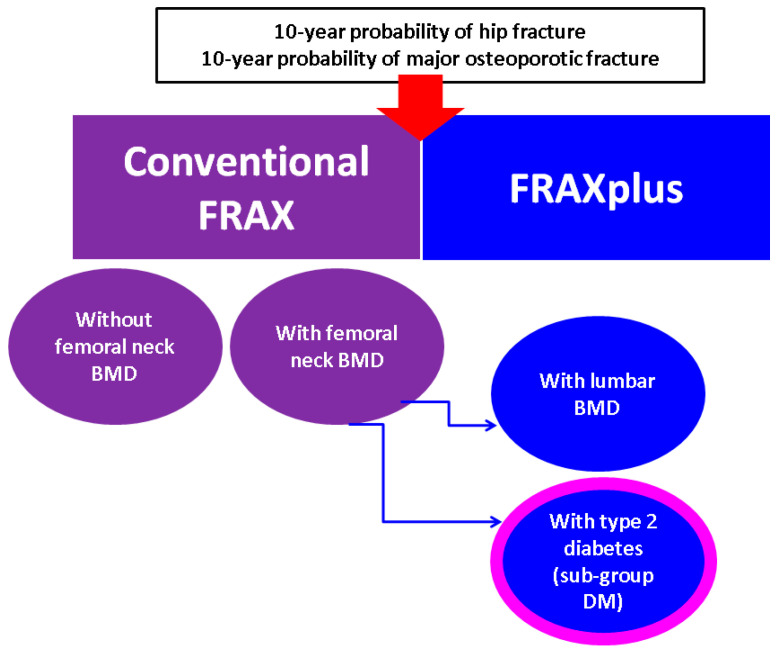
10-year probability of hip and major osteoporotic fracture assessments according to the conventional FRAX [13] and FRAXplus [16].

The estimation of fracture probability was provided by the FRAX model according to the conventional FRAX estimates [13] and the FRAXplus model, respectively. The FRAXplus model estimates were performed with adjustments for type 2 diabetes and lumbar BMD [16]. Either model provided MOF and HF (Figure 2).

**Ethical aspects**: The retrospective study was approved by the local Ethical Committe (number 702 from 28 June 2024; number 127 from 25 June 2024; number 6284 from 8 February 2024).

**Statistical analysis**: Kolmogorov-Smirnov test was applied to determine the normality of the data distribution. Descriptive statistics were calculated using the mean ± standard deviation (SD) for normally distributed data or the median and quartiles Q1, Q3 for non-normally distributed data. The t-test was used to compare the means of two independent groups to determine if there was a statistically significant difference between them. The Mann-Whitney U test was employed to compare differences between two independent groups when the data did not meet the assumptions of normality. To control for potential confounding by BMI, when comparing the DM and nonDM subgroups we performed multivariable linear regression analyses for each continuous parameter. In these models, the parameter of interest was used as the dependent variable (Y), while diabetes status was included as the main independent variable (X_1_), and BMI was added as a covariate (X_2_). This approach allowed for the assessment of the independent association between diabetes and the variable of interest, adjusting for the potential confounding effect of BMI. Adjusted *p*-values were derived from these regression models to complement the unadjusted comparisons and to improve the validity of between-group inferences. Bivariate correlation analysis was performed to assess the strength and direction of the linear relationship between two continuous variables. Pearson’s correlation coefficient was used for normally distributed data and Kendall’s Tau rank correlation coefficient was used for non-normally distributed data. Multiple regression analysis was employed to examine the relationship between a dependent variable and multiple independent variables. Regression coefficients ± standard error (B ± SE) showed the change in the dependent variable for a one-unit change in the corresponding independent variable, holding all other variables constant. Standardized coefficients (β weights) were used to compare the relative importance of the independent variables. R^2^ showed the explained variance by the computed model. The statistical analysis of the data was performed using the following software packages: Excel 16.93 (Microsoft, Redmond, WA, USA) and SPSS 29.0.2.0 (SPSS, Inc., Chicago, IL, USA).

## 3. Results

### 3.1. DXA and BTM Analysis

A total of 136 menopausal patients were analyzed, with a mean age of 61.36 ± 8.20 years and 14.60 ± 9.21 years since menopause; average BMI was 27.71 ± 5.42 kg/m^2^. Of the entire cohort, 22.06% of the patients were sub-group DM versus sub-group nonDM (N = 106, 77.94%). Age and years since menopause were similar between the sub-groups, as well as the percent of prevalent osteoporotic fractures.

BMI was statistically significantly higher in sub-group DM (31.80 ± 5.31 kg/m^2^) compared to group nonDM (26.54 ± 4.87 kg/m^2^; *p* < 0.001), as well as glycated hemoglobin A1c (6.59 ± 1.25% versus 5.49 ± 0.41%; *p* < 0.001). After adjusting for BMI, glycated hemoglobin A1c remained statistically significantly higher in sub-group DM versus nonDM (*p* < 0.001) (Table 1).

**Figure 3:**
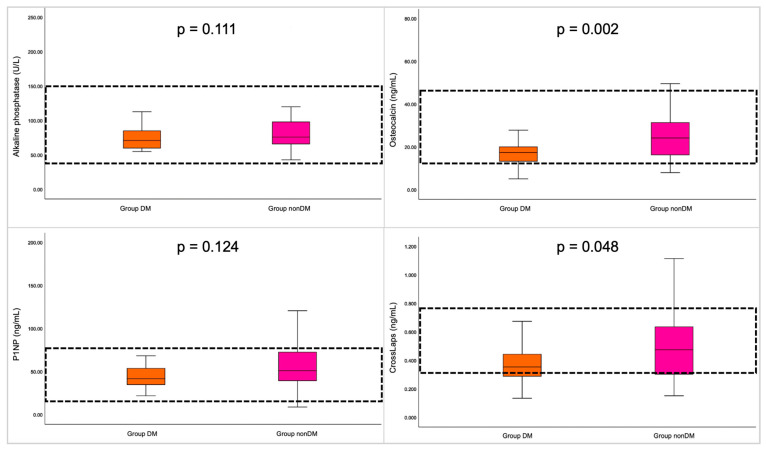
Boxplots showing BTM distribution in studied subgroups.

Serum total and ionized serum calcium, phosphorus, and magnesium were similar among the two sub-groups. Osteocalcin (18.09 ± 8.35 ng/mL) in sub-group DM was found statistically significantly lower versus sub-group nonDM (25.62 ± 12.78 ng/mL; *p* = 0.002), as well as CrossLaps (0.39 ± 0.18 ng/mL versus 0.48 ± 0.22 ng/mL; *p* = 0.048). The other two bone formation markers, namely, alkaline phosphatase and P1NP, were similar between the sub-groups. However, after BMI adjustment, alkaline phosphatase was lower in diabetic versus non-diabetic females (*p* = 0.024). 25-hydroxyvitamin D was lower in sub-group DM versus nonDM (*p* = 0.013) (Table 2, Figure 3).

Lumbar BMD and T-score were similar between the groups. Femoral neck BMD/T-score were statistically significantly higher in sub-group DM versus nonDM (*p* = 0.007, respectively *p* = 0.002), as well as total hip BMD/T-score (*p* = 0.002 for each).

### 3.2. Multiple Linear Regression Models for BMD at Central DXA

The regression model explained 42.7% of the variance in lumbar BMD (R^2^ = 0.427). The baseline lumbar BMD in the absence of other predictors was of 1.367 ± 0.287 g/sqcm (*p* < 0.001). Age showed a statistically significant influence on lumbar BMD, indicating that an increase in age with 1 year corresponded to a decrease in lumbar BMD with −0.012 ± 0.003 g/sqcm (*p* < 0.001). BMI was also a statistically significant contributor to lumbar BMD: an increase in BMI with 1 kg/m^2^ led to an increase in lumbar BMD of 0.012 ± 0.004 g/sqcm (*p* < 0.001). The presence of DM, osteocalcin and 25-hydroxyvitamin D was not statistically significantly correlated with lumbar BMD. Age was the most influential contributor in the model, with the highest β of −0.475 (Table 3).

The multiple linear regression model explained 60.0% of the variation in femoral neck BMD (R^2^ = 0.600). Baseline femoral neck BMD when all contributors were 0 was 0.768 ± 0.170 g/sqcm (*p* < 0.001). The presence of DM led to a statistically significant increase in femoral neck BMD of 0.079 ± 0.036 g/sqcm (*p* = 0.030). BMI increase of 1 kg/m^2^ and 25-hydroxyvitamin D of 1 ng/mL were also statistically significantly correlated to femoral neck BMD increase of 0.009 ± 0.003 g/sqcm (*p* < 0.001) and, respectively of 0.004 ± 0.001 g/sqcm (*p* = 0.007). Age and CrossLaps were negatively correlated with femoral neck BMD as followings: increase of age with 1 year led to decrease of BMD with −0.006 ± 0.002 g/sqcm (*p* < 0.001) and increase in CrossLaps with 1 ng/mL led to decrease of BMD with −0.204 ± 0.080 g/sqm (*p* = 0.013). Highest β was for BMI of 0.375, stating that BMI was the strongest predictor for femoral neck BMD in this model (Table 4).

The multivariate linear regression model for total hip BMD explained the variation of 65.0% (R^2^ = 0.650). Total hip BMD value in the absence of other predictors was 0.899 ± 0.190 g/sqcm (*p* < 0.001). The presence of DM was a positive predictor for total hip BMD, resulting in an increase of 0.079 ± 0.039 g/sqcm (*p* = 0.048), as well as BMI and 25-hydroxyvitamin D [increase of 0.012 ± 0.003 g/sqcm for 1 kg/m^2^ (*p* < 0.001), respectively, of 0.003 ± 0.002 g/sqcm for 1 ng/mL (*p* = 0.044)]. Age and CrossLaps were statistically significant negative contributors to total hip BMD, considering that 1 year increase in age led to a decrease of −0.007 ± 0.002 g/sqcm in total hip BMD and 1 ng/mL increase in CrossLaps led to a decrease of −0.230 ± 0.089 g/sqcm decrease in total hip BMD. BMI had the highest effect on total hip BMD in this model, with the highest β of 0.434 (Table 5).

### 3.3. The Analysis of 10-Year Fracture Probability

MOF adjusted for lumbar BMD (FRAXplus) in entire cohort was statistically significantly lower compared to MOF estimated without femoral neck BMD (FRAX) (*p* < 0.001), respectively MOF calculated with femoral neck BMD (FRAX) (*p* < 0.001) (Table 6).

**Figure 4:**
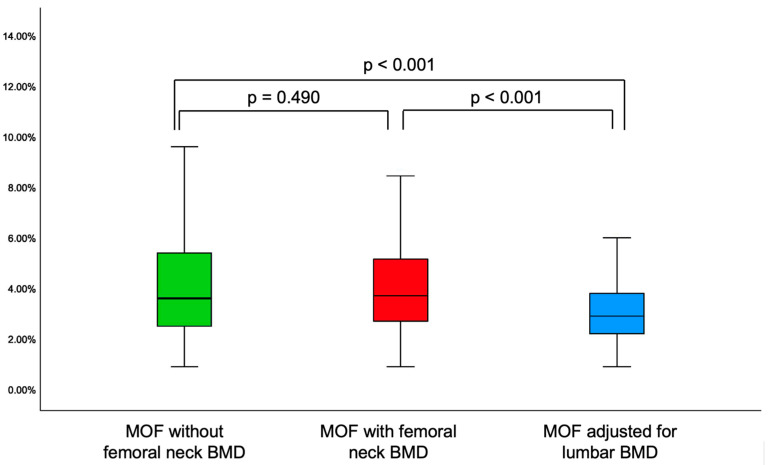
Whisker plots showing the distribution of 10-year probability for major osteoporotic fractures (MOF).

MOF without femoral neck BMD (FRAX) and MOF with femoral neck BMD (FRAX) were similar (*p* = 0.490) (Figure 4).

HF adjusted for lumbar BMD (FRAXplus) was statistically significantly lower versus HF without femoral neck BMD (FRAX) (*p* = 0.008), respectively, HF with femoral neck BMD (FRAX) (*p* < 0.001) (Table 7).

**Figure 5:**
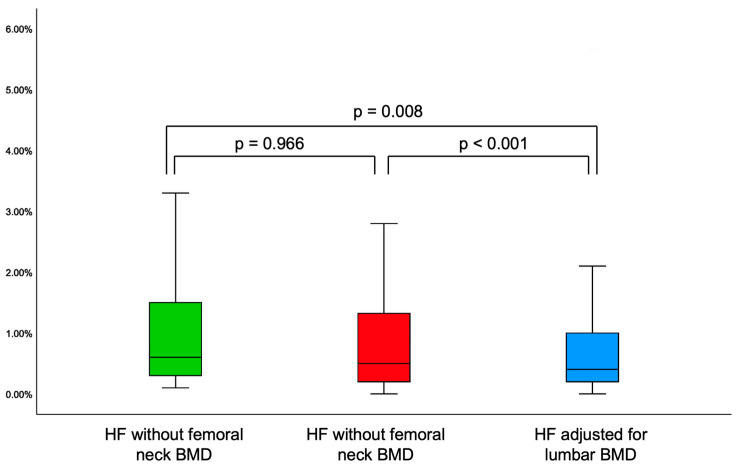
Whisker plots showing the distribution of 10-year probability for hip fracture (HF).

HF without femoral neck BMD (FRAX) and HF with femoral neck BMD (FRAX) were similar (*p* = 0.966) (Figure 5).

HF calculated with femoral neck BMD (FRAX) was statistically significantly lower in sub-group DM versus sub-group nonDM (*p* = 0.027), as well as HF adjusted for lumbar BMD (FRAXplus) (*p* = 0.007). However, after adjusting for BMI, all estimated fracture risks were similar between the mentioned sub-groups (Table 8).

Sub-group DM had a MOF adjusted for lumbar BMD (FRAXplus) statistically significantly lower versus MOF without, respectively, with femoral neck BMD (FRAX) (*p* < 0.001 for each), and lower versus MOF adjusted for the duration of type 2 diabetes (FRAXplus) (*p* < 0.001). On the other hand, MOF adjusted for diabetes was statistically significantly higher versus MOF with femoral neck BMD (FRAX) (*p* < 0.001) (Table 9).

**Figure 6:**
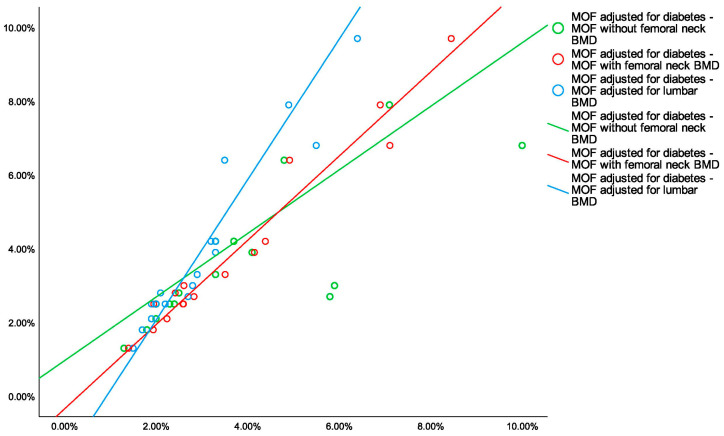
Scatterplot showing the correlation between 10-year probabilities of major osteoporotic fractures (MOF) adjusted for type 2 diabetes and the other MOFs in diabetic menopausal subjects (BMD = bone mineral density).

The correlation analysis in diabetic females (sub-group DM) showed that MOF without femoral neck BMD (FRAX), MOF with femoral neck BMD (FRAX), MOF adjusted for lumbar BMD (FRAXplus), and MOF adjusted for type 2 diabetes (FRAX) were all positively correlated. The highest correlation coefficient was between MOF adjusted for lumbar BMD (FRAXplus) and MOF adjusted for type 2 diabetes (FRAXplus) (r = 0.927, *p* < 0.001). The lowest correlation coefficient was between MOF without femoral neck BMD (FRAX) and MOF with femoral neck BMD (FRAX) (r = 0.711, *p* < 0.001). (Table 10, Figure 6)

In diabetic subgroup, 10-year probability for hip fracture (FRAXplus) adjusted for lumbar BMD was statistically significantly lower versus HF without femoral neck BMD (FRAX) (*p* < 0.001), respectively, HF with femoral neck BMD (FRAX) (*p* < 0.001), as well as HF adjusted for the duration of type 2 diabetes (FRAXplus) (*p* < 0.001). Moreover, HF adjusted for the diabetes (FRAXplus) was statistically significant higher versus HF calculated with femoral neck BMD (FRAX) (*p* < 0.001) (Table 11).

**Figure A1:**
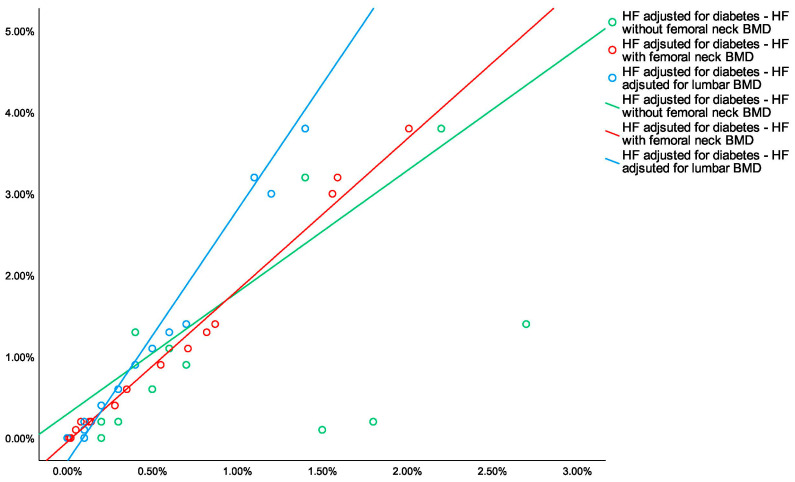
Scatterplot showing the correlation between 10-year probability for hip fracture adjusted for type 2 diabetes and the other HFs in diabetic subjects.

In type 2 diabetic subjects, statistically significant and positive correlations were found with respect to all four 10-year probabilities of hip fracture [the strongest correlation was between HF with femoral neck BMD and HF adjusted for type 2 diabetes (r = 0.961, *p* < 0.001)] (Table 12; Figure A1 Appendix A).

In diabetic subjects, the multiple linear regression models had an R^2^ of 0.986 that explained 98.6% of the variation of MOF adjusted for type 2 diabetes [a statistically significance was found for MOF calculated with femoral neck BMD (*p* = 0.025)]. HF had an R^2^ of 0.999, explaining 99.9% of the variation of HF adjusted for diabetes (*p* < 0.001 for HF with femoral neck BMD) (Table 13).

In non-diabetic subjects, MOF adjusted for lumbar BMD (FRAXplus) was statistically significantly lower versus MOF without femoral neck BMD (*p* < 0.001), respectively, MOF with femoral neck BMD (*p* < 0.001). HF adjusted for lumbar BMD (FRAXplus) was statistically significantly lower versus HF without femoral neck BMD (*p* < 0.001) and HF with femoral neck BMD (*p* < 0.001) (Table A1 Appendix B).

## 4. Discussion

Type 2 diabetes represents a distinct category in the large panel of fracture risk contributors and multidisciplinary causes of secondary osteoporosis [20,21,22,23,24]. In this observational study, we analyzed two menopausal sub-groups across a cohort with an average age of 61.36 years; the sub-groups of type 2 diabetic versus non-diabetic females showed a similar age, and duration of post-menopause (the entire cohort had a mean 14.6 menopause-years). However, sub-group DM displayed a **higher BMI**, as expected, and we provided a BMI adjustment for the statistical analysis. Generally, obesity in menopausal subjects might increase the fracture risk in certain sites, independently or across the confirmation of metabolic syndrome, as seen in other associated ailments [25,26,27,28]. Yet, an increased BMI correlates with an elevated BMD at DXA, thus, an underestimation of the fracture risk assessment might come by only using the central DXA evaluation in obese individuals, and tools such as FRAX/FRAXplus might bridge this gap in every day practice [29,30,31].

The studied sub-groups had a similar rate of prevalent **osteoporotic fractures**, twelve out of 136 individuals of the entire study population had an osteoporotic fracture, but they were no exposed at any point in life to any specific drug against osteoporosis. This is not unusual, noting this was a real-life study, and the patients were not pre-selected. For instance, we mention a recent meta-analysis and systematic review which has been published in 2025 showing that people diagnosed with type 2 diabetes have a higher relative risk of osteoporosis (of 1.841, 95% confidence interval between 1.219 and 2.78, *p* = 0.004) versus non-diabetic population, and a similar increase of osteoporotic fracture risk (relative risk of 1.21, 95% confidence interval between 1.09 and 1.31, *p* < 0.001). Mostly important, we should mention that Cao et al. [3] did not confirm this higher risk in certain sub-groups of analysis that referred, among others, to cross-sectional studies, as the current one, neither by only applying a univariate regression, nor in certain population groups. This highlights the fact that multiple heterogeneous aspects are still an open issue in understanding and assessing the osteoporotic fracture risk in type 2 diabetic subjects [3]. Similarly, Wenhao et al. did not show a causal relationship between osteoporosis onset and progression (including complicated with fractures) and the confirmation of type 2 diabetes [32].

In addition, recent results from the PARADOS study [33], also a cross-sectional cohort in type 2 diabetic, postmenopausal women, showed that the relationship between diabetes and the prevalence of osteoporosis, respectively between diabetes and sarcopenia was inverse, meaning that diabetes was a protective factor for osteoporosis (OR: 0.477, 95% CI 0.310–0.733) and an independent risk factor for sarcopenia (OR: 1.887, 95% CI 1.107–3.218) [33]. These results may be highly variable with the particular features of the enrolled population since diabetic bone disease represents a multifactorial constellation [34]. For example, in current study, we analyzed a population with a total prevalence of osteoporosis (based on lowest T-score at central DXA) of 22.06%, with similar rates between the studied sub-groups of 16.67% (DM) versus 23.58% (nonDM), while Tiftik et al. have found an osteoporosis frequency of 33.5% in 158 diabetic females [33]. Notably, Liu et al. [35] showed a similar rate across a meta-analysis and systematic review from 2023, namely, of 27.67% (95% confidence interval between 21.37 and 33.98%). Across 21 observational studies, 11,603 patients diagnosed with type 2 diabetes were included, but, overall, a significant increased heterogeneity was observed among the analyzed cohorts (I^2^ = 98.5%) [35].

Another underlying pathway between type 2 diabetes and fractures may be a chronic low-grade inflammation that also targets the bone [36,37,38,39]. A recent study in menopausal diabetic subjects revealed that systemic immune-inflammatory index inversely correlated with BMD at DXA and positively associated with the 10-year probability of major osteoporotic fractures, respectively, hip fracture. The patients with a high index (≥629.46, N = 141/423) showed the most elevated fracture risk (*p* = 0.011). Interestingly, the co-presence of anemia increased the risk of fracture by 4.5 times (*p* = 0.01) [40], and this might represent a future expansion of our study.

In this study, **multiple regression models** confirmed the age- and BMI-related influence on lumbar DXA-BMD, while the confirmation that the patient was type 2 diabetic or the serum levels of osteocalcin and 25-hydroxyvitamin D did not influence the lumbar BMD. On the other hand, type 2 diabetes statistically significant increased femoral neck BMD, the same as BMI increase and 25-hydroxyvitamin D increases, while age and CrossLaps was negatively correlated with femoral neck BMD. The multivariate logistic regression model in total hip BMD explained the variation of 65%; diabetic disease being a positive predictor, as found for BMI and 25-hydroxyvitamin D; age and CrossLaps displayed a negative influence. According to our model, the strongest predictor remained BMI with concern to the femoral neck and total hip BMD. As mentioned, the relationship between reduced BMD at DXA and type 2 diabetes is not linear, if any; for instance, a meta-analysis published by Qiu et al. in 2021 found (across 14 studies, N = 24,340 individuals) no such relationship [41]. Hence, the need and the importance of developing practical algorithms/models to assess the osteoporotic fracture risk in daily practice from a multidisciplinary perspective.

**Current tools** to address the fracture risk estimation in type 2 diabetes underestimate it and new models are under development [16]. We mention a similar study in 107 menopausal women with type 2 diabetes and among those who did not receive bone-active drugs (despite high risk) for a median follow-up of 60.2 months, 13% experienced an incidental fragility fracture [7]. This means that pinpointing an increased fracture risk helps an early selection of the patients who are suitable candidates to medication for fracture risk reduction. In this study, we found across the entire cohort of menopausal females, that MOF, respectively, HF adjusted for lumbar BMD (FRAXplus) was statistically significantly lower compared to the risks estimated without femoral neck BMD (FRAX) with *p* < 0.001, respectively, *p* = 0.008, or calculated with femoral neck BMD (FRAX) (*p* < 0.001 for each). In the diabetic sub-groups, the same results were applied (*p* < 0.001 for each); however, when estimating the risk with type 2 diabetes adjustment, MOFs was higher than MOF calculated with femoral neck BMD (*p* < 0.001), hence, confirming that the other risk calculations that do not consider the presence/duration of diabetes might underestimate the risk. Overall, we identified a tight correlation between any of the four calculated probabilities, suggesting that in low-risk populations, as seen here, no major discrepancies in risk estimation are expected. Moreover, in diabetic subjects, the multiple linear regression models had an R^2^ of 0.986 that explained 98.6% of the variation of MOF adjusted for diabetes [a statistically significance was found for MOF calculated with femoral neck BMD (*p* = 0.025)], while HF had an R^2^ of 0.999, explaining 99.9% of the variation of HF adjusted for diabetes (*p* < 0.001 for HF with femoral neck BMD).

Overall, FRAXplus might prevail over the **limitations** of the current risk calculation and further implementation on a larger scale is needed [35,36,37,38,39,40,41,42]. To our best knowledge, this study is one of the earliest that has been introduced so far with respect to the novel adjustments such as the type 2 diabetes or the use of lumbar BMD according to central DXA. As limits of the study we mention the followings, in addition to the retrospective, transversal design and the sample size. We did not quantify the vitamin D and calcium supplementation in the study population, but provided the blood mineral metabolism assays in terms of calcemic level and 25-hydroxyvitamin D. While serum nutrients were similar between the sub-groups, vitamin D status was more affected in diabetic females with statistically significant lower values of 25-hydroxyvitamin D (16.96 ± 6.76 versus 21.29 ± 9.84 ng/mL, *p* = 0.013). Notably, vitamin D receptor gene polymorphism might be one of the signal transduction pathways for developing diabetic bone disease [4]. Over the latest decades, nutritional role had been linked to the skeletal health in various ways [43,44,45,46,47,48,49,50]. We did not analyze the type of fractures because of their relative small number (N = 12). None of them was a hip fracture and the overall studied cohort displayed a relatively low 10-year probability of any fracture. While some authors showed certain associations between fasting glycaemia and BMD at DXA [2], we appreciated that glucose might suffer daily fluctuation that were out of our scope. Generally, the glucose profile is considered a reflection of the oxidative stress status, advanced glycation end products, as well as the inflammatory spectrum, and all of them might be prone to negatively interact with the bone cells and the mineralization process that ensures the bone strength [2]. Of note, FRAXplus takes into consideration type 2 diabetes duration, not the disease control (e.g., glycated hemoglobin A1c) and further studies are useful. Finally, we excluded the patients who were treated with insulin therapy, noting that this regime has been proved to particularly increase the fracture risk in middle-aged and seniors type 2 diabetic individuals [51]. The insulin therapy represents a proved risk factor by itself to increase the fracture risk, and, by excluding it, we intended to capture the essence of the diabetes, not of the associated medication. With respect to novel agents in the field of type 2 diabetes there been conflicting results so far, and by excluding them, we intended to reduce the iatrogenic bias. Further research will pinpoint their impact on the bone health. Also, we should take into consideration a (potential) minor impact of the diabetic bone disease might have had on the analysis in view of its good control as reflected by the values of A1c glycated hemoglobin (an average of 6.5%). However, diabetes duration and its potential effects on the bone health were captured by novel FRAX algorithm with adjustments for the type 2 diabetic adults. Also, we point out the importance of using and expanding the model risk prediction algorithms from a cost-effectiveness perspective and clinical purposes in the everyday practice, especially in resource-limited settings, and FRAXplus might address the type 2 diabetes-related fracture risk from this point of view. Moreover, the data in diabetic population may be expanded to novel fracture risk estimators such as Fragility Score based on Radiofrequency Echographic Multi Spectometry (REMS)-based indicator for the prediction of incident fractures [52], as well as prospective long-term studies to address the incident fractures as primary outcome.

## 5. Conclusions

🟩 Noting the epidemiologic impact of type 2 diabetes, and the importance of the diabetic bone disease, particularly, from a practical perspective, the osteoporotic fracture risk estimation might help the overall disease burden. New algorithms such as FRAXplus are in progress to help this distinct matter.
🟩 In this study, type 2 diabetic menopausal women when compared to age- and years since menopause-match controls had a lower 25-hydroxyvitamin D and BTMs (osteocalcin, CrossLaps), an increased total hip BMD and femoral neck BMD (with loss of significance upon BMI adjustment).
🟩 When applying novel FRAX model, lumbar spine BMD adjustment showed lower MOF and HF as estimated by the conventional FRAX (in either subgroup or entire cohort) or as found by diabetes adjustment using FRAXplus (in diabetic subgroup).
🟩 To date, all four types of 10-year fracture probabilities displayed a strong correlation, but taking into consideration the presence of the diabetic disease, statistically significant higher risks than calculated by the traditional FRAX were found, hence, the current model might underestimate the condition-related fracture risk.
🟩 Addressing the practical aspects of fracture risk assessment in diabetic menopausal women might improve the bone health and further offers a prompt tailored strategy to reduce the fracture risk.

## Figures and Tables

**Table 1:** Demographic characteristics of the entire group (N = 136), subgroup DM andnonDM.

Parameter	Entire Group (N = 136, 100%)	Group DM (N = 30, 22.06%)	Group nonDM (N = 106, 77.94%)	*p*-Value	*p*-Value Adjusted for BMI
Age (years), mean ± SD	61.36 ± 8.20	61.87 ± 7.62	61.22 ± 8.39	0.703	0.298
Years since menopause, mean ± SD	14.60 ± 9.21	15.13 ± 7.82	14.45 ± 9.60	0.722	0.445
BMI (kg/m^2^), mean ± SD	27.71 ± 5.42	31.80 ± 5.31	26.54 ± 4.87	**<0.001**	N/A
Prevalent fractures, N (%)	12 (8.76)	2 (6.66)	10 (9.43)	0.737	0.982
Dyslipidemia, N (%)	85 (62.50)	24 (80.00)	61 (57.55)	**0.025**	0.159
Glycated hemoglobin A1c (%), mean ± SD *	5.82 ± 0.90	6.59 ± 1.25	5.49 ± 0.41	**<0.001**	**<0.001**
Normal DXA, N (%)	38 (27.94)	13 (43.33)	25 (23.58)	**0.040**	0.440
Osteopenia, N (%)	68 (50.00)	12 (40.00)	56 (52.83)	0.215	0.180
Osteoporosis, N (%)	30 (22.06)	5 (16.67)	25 (23.58)	0.420	0.380
Less than 5 years, N (%) **		18 (60.00)			
Between 5 and 10 years, N (%) **		9 (30.00)			
More than 10 years, N (%) **		3 (10.00)			

Abbreviations: BMI = body mass index, N/A = not applicable, N = number of patients, SD = standard deviation; * normal range: 4.8–5.9%; ** duration of type 2 diabetes (sub-group DM).

**Table 2:** Bone health parameters in the entire cohort, sub-group DM and nonDM.

Parameter	Normal Range	Entire Cohort (N = 136, 100%)	Sub-Group DM (N = 30, 22.06%)	Sub-Group nonDM (N = 106, 77.94%)	*p*-Value	*p*-Value Adjusted for BMI
**Mineral metabolism**						
Total serum calcium (mg/dL), mean ± SD	8.4–10.3	9.57 ± 0.55	9.67 ± 0.44	9.55 ± 0.58	0.291	0.624
Ionized serum calcium (mg/dL), mean ± SD	3.9–4.9	4.14 ± 0.32	4.20 ± 0.19	4.13 ± 0.34	0.512	0.802
Total proteins (g/dL), mean ± SD	6.4–8.6	7.39 ± 0.49	7.44 ± 0.52	7.38 ± 0.48	0.594	0.719
Serum phosphorus (mg/dL), mean ± SD	2.5–4.5	3.68 ± 0.58	3.57 ± 0.43	3.71 ± 0.62	0.293	0.655
Serum magnesium (mg/dL), mean ± SD	1.6–2.6	1.97 ± 0.19	1.90 ± 0.27	1.99 ± 0.16	0.167	0.220
25-hydroxyvitamin D (ng/mL), mean ± SD	30–100	20.39 ± 9.43	16.96 ± 6.76	21.29 ± 9.84	**0.013**	0.161
PTH (pg/mL), mean ± SD	16–65	50.63 ± 24.38	49.26 ± 24.23	51.05 ± 24.58	0.759	0.851
**Bone turnover markers**						
Osteocalcin (ng/mL), mean ± SD	15–46	23.97 ± 12.32	18.09 ± 8.35	25.62 ± 12.78	**0.002**	0.070
Alkaline phosphatase (U/L), mean ± SD	40–150	83.14 ± 32.60	74.21 ± 18.54	85.87 ± 35.46	0.111	**0.024**
P1NP (ng/mL), mean ± SD	20.25–76.31	55.17 ± 30.13	44.30 ± 16.41	58.48 ± 32.62	0.124	0.193
CrossLaps (ng/mL), mean ± SD	0.33–0.782	0.46 ± 0.21	0.39 ± 0.18	0.48 ± 0.22	**0.048**	0.232
**DXA evaluation**						
Lumbar BMD (g/sqcm), mean ± SD		1.025 ± 0.192	1.042 ± 0.262	1.020 ± 0.168	0.597	0.201
Lumbar T-score (SD), mean ± SD	>−1	−1.17 ± 1.42	−0.75 ± 1.51	−1.29 ± 1.38	0.069	0.913
Lumbar Z-score (SD), mean ± SD		−0.26 ± 1.23	−0.06 ± 1.35	−0.31 ± 1.19	0.330	0.398
Femoral neck BMD (g/sqcm), mean ± SD		0.872 ± 0.144	0.934 ± 0.154	0.854 ± 0.136	**0.007**	0.509
Femoral neck T-score (SD), mean ± SD	>−1	−1.12 ± 1.00	−0.61 ± 1.61	−1.26 ± 0.91	**0.002**	0.244
Femoral neck Z-score (SD), mean ± SD		−0.01 ± 0.85	0.29 ± 1.09	−0.09 ± 0.76	0.086	0.145
Total hip BMD (g/sqcm), mean ± SD		0.947 ± 0.160	1.031 ± 0.170	0.924 ± 0.150	**0.002**	0.282
Total hip T-score (SD), mean ± SD	>−1	−0.47 ± 1.25	0.20 ± 1.35	−0.65 ± 1.17	**0.002**	0.277
Total hip Z-score (SD), mean ± SD		0.33 ± 1.01	0.81 ± 1.14	0.19 ± 0.94	**0.005**	0.104

Abbreviations: BMI = body mass index, BMD = bone mineral density, DXA = Dual-Energy X-Ray Absorptiometry, N = number of patients, SD = standard deviation.

**Table 3:** Multiple linear regression model to predict lumbar BMD at central DXA.

	Lumbar BMD		
Parameter	B ± SE	β	*p*-Value
Constant	1.367 ± 0.287		**<0.001**
Type 2 diabetes mellitus	−0.037 ± 0.060	−0.077	0.544
Age	−0.012 ± 0.003	−0.475	**<0.001**
Body mass index	0.012 ± 0.004	0.336	**0.009**
Osteocalcin	−0.002 ± 0.002	−0.094	0.522
CrossLaps	−0.131 ± 0.135	−0.134	0.336
25-hydroxyvitamin D	0.003 ± 0.003	0.122	0.255
	R^2^ = 0.427		

Abbreviations: BMD = bone mineral density, B = unstandardized regression coefficient, SE = standard error, β = standardized regression coefficient, R^2^ = multiple correlation coefficient.

**Table 4:** Multiple linear regression model to predict femoral neck BMD.

	Femoral Neck BMD		
Parameter	B ± SE	β	*p*-Value
Constant	0.768 ± 0.170		**<0.001**
Type 2 diabetes mellitus	0.079 ± 0.036	0.233	**0.030**
Age	−0.006 ± 0.002	−0.342	**<0.001**
Body mass index	0.009 ± 0.003	0.375	**<0.001**
Osteocalcin	0.001 ± 0.001	0.093	0.451
CrossLaps	−0.204 ± 0.080	−0.296	**0.013**
25-hydroxyvitamin D	0.004 ± 0.001	0.246	**0.007**
	R^2^ = 0.600		

Abbreviations: BMD = bone mineral density, B = unstandardized regression coefficient, SE = standard error, β = standardized regression coefficient, R^2^ = multiple correlation coefficient.

**Table 5:** Multiple linear regression model to predict total hip BMD.

	Total Hip BMD		
Parameter	B ± SE	β	*p*-value
Constant	0.899 ± 0.190		**<0.001**
Type 2 diabetes mellitus	0.079 ± 0.039	0.209	**0.048**
Age	−0.007 ± 0.002	−0.367	**<0.001**
Body mass index	0.012 ± 0.003	0.434	**<0.001**
Osteocalcin	0.001 ± 0.002	0.048	0.693
CrossLaps	−0.230 ± 0.089	−0.298	**0.012**
25-hydroxyvitamin D	0.003 ± 0.002	0.177	**0.044**
	R^2^ = 0.650		

Abbreviations: BMD = bone mineral density, B = unstandardized regression coefficient, SE = standard error, β = standardized regression coefficient, R^2^ = multiple correlation coefficient.

**Table 6:** Related samples test for equality of MOF distribution (BMD = bone mineral density, Q = quartile).

10-Year Probability of Major Osteoporotic Fracture (%)	Value
without femoral neck BMD, median (Q1, Q3)	3.70 (2.50, 5.65)
with femoral neck BMD, median (Q1, Q3)	3.70 (2.10, 5.40)
adjusted for lumbar BMD, median (Q1, Q3)	2.90 (2.20, 3.80)

**Table 7:** Related samples test for equality of HF distribution (BMD = bone mineral density, Q = quartile).

10-Year Probability of Hip Fracture (%)	Value
without femoral neck BMD, median (Q1, Q3)	0.60 (0.30, 160)
with femoral neck BMD, median (Q1, Q3)	0.50 (0.20, 1.40)
adjusted for lumbar BMD (%), median (Q1, Q3)	0.40 (0.20, 1.00)

**Table 8:** Analysis of 10-year probability of fracture in diabetic versus non-diabetic females (BMI = body mass index, BMD = bone mineral density, MOF = 10-year fracture risk of major osteoporotic fracture, HF = 10-year fracture risk of hip fracture, Q = quartile, N = number of patients).

10-Year Probability of Fracture (%)	Sub-Group DM (N = 30)	Sub-Group nonDM (N = 106)	*p*-Value	*p*-Value Adjusted for BMI
MOF without femoral neck BMD, median (Q1, Q3)	3.40 (2.10, 5.80)	3.80 (2.70, 5.50)	0.306	0.377
MOF with femoral neck BMD, median (Q1, Q3)	3.10 (2.30, 4.39)	3.90 (2.90, 5.63)	0.078	0.735
MOF adjusted for lumbar BMD, median (Q1, Q3)	2.75 (1.90, 3.25)	3.00 (2.30, 4.30)	0.121	0.705
MOF adjusted for type 2 diabetes, median (Q1, Q3)	3.70 (2.50, 5.60)			
HF without femoral neck BMD, median (Q1, Q3)	0.50 (0.20, 1.50)	0.60 (0.40, 1.70)	0.191	0.422
HF with femoral neck BMD, median (Q1, Q3)	0.35 (0.13, 0.80)	0.59 (0.30, 1.53)	**0.027**	0.792
HF adjusted for lumbar BMD, median (Q1, Q3)	0.20 (0.10, 0.45)	0.40 (0.20, 1.10)	**0.007**	0.959
HF adjusted for type 2 diabetes, median (Q1, Q3)	0.80 (0.20, 2.40)			

**Table 9:** Related samples test for equality of MOF distribution in sub-group DM (BMD = bone mineral density, Q = quartile).

10-Year Probability of Major Osteoporotic Fractures (%)	Value
without femoral neck BMD, median (Q1, Q3)	3.40 (2.10, 5.80)
with femoral neck BMD, median (Q1, Q3)	3.10 (2.30, 4.39)
adjusted for lumbar BMD, median (Q1, Q3)	2.75 (1.90, 3.25)
adjusted for type 2 diabetes, median (Q1, Q3)	3.70 (2.50, 5.60)

**Table 10:** Correlation coefficients between 10-year probability of major osteoporotic fractures in diabetic females (BMD = bone mineral density).

10-Year Probability for Major Osteoporotic Fracture	Without Femoral Neck BMD	With Femoral Neck BMD	Adjusted for Lumbar BMD	Adjusted for Type 2 Diabetes
without femoral neck BMD		r = 0.711 ***p* < 0.001**	r = 0.769 ***p* < 0.001**	r = 0.740 ***p* < 0.001**
with femoral neck BMD	r = 0.711 ***p* < 0.001**		r = 0.923 ***p* < 0.001**	r = 0.908 ***p* < 0.001**
adjusted for lumbar BMD	r = 0.769 ***p* < 0.001**	r = 0.923 ***p* < 0.001**		r = 0.927 ***p* < 0.001**
adjusted for type 2 diabetes	r = 0.740 ***p* < 0.001**	r = 0.908 ***p* < 0.001**	r = 0.927 ***p* < 0.001**	

**Table 11:** Related samples test for equality of 10-year probability of hip fracture distribution in diabetic females (BMD = bone mineral density, Q = quartile).

10-Year Probability of Hip Fracture (%)	Value
without femoral neck BMD (%), median (Q1, Q3)	0.50 (0.20, 1.50)
with femoral neck BMD (%), median (Q1, Q3)	0.35 (0.13, 0.80)
adjusted for lumbar BMD (%), median (Q1, Q3)	0.20 (0.10, 0.45)
adjusted for diabetes (%), median (Q1, Q3)	0.80 (0.20, 2.40)

**Table 12:** Correlations coefficients between 10-year probability of hip fracture in diabetic females (BMD = bone mineral density).

10-Year Probability of Hip Fracture	Without Femoral Neck BMD	With Femoral Neck BMD	Adjusted for Lumbar BMD	Adjusted for Type 2 Diabetes
without femoral neck BMD		r = 0.478 ***p* < 0.001**	r = 0.573 ***p* < 0.001**	r = 0.570 ***p* = 0.001**
with femoral neck BMD	r = 0.478 ***p* < 0.001**		r = 0.856 ***p* < 0.001**	r = 0.961 ***p* < 0.001**
adjusted for lumbar BMD	r = 0.573 ***p* < 0.001**	r = 0.856 ***p* < 0.001**		r = 0.942 ***p* < 0.001**
adjusted for diabetes	r = 0.570 ***p* = 0.001**	r = 0.961 ***p* < 0.001**	r = 0.942 ***p* < 0.001**	

**Table 13:** Multiple linear regression model to show interactions between MOFs, respectivel, HFs in diabetic females (BMD = bone mineral density, MOF = 10-year fracture risk of major osteoporotic fracture, HF = 10-year probability of hip fracture; B = unstandardized regression coefficient, SE = standard error, β = standardized regression coefficient, R^2^ = multiple correlation coefficient).

	**MOF Adjusted for Diabetes**		
**Parameter**	**B ± SE**	**β**	***p*-Value**
Constant	0.272 ± 0.725		0.713
MOF without femoral neck BMD	0.139 ± 0.181	0.096	0.454
MOF with femoral neck BMD	1.756 ± 0.700	1.526	**0.025**
MOF adjusted for lumbar BMD	−1.163 ± 1.334	−0.598	0.398
	R^2^ = 0.986		
	**HF Adjusted for Diabetes**		
**Parameter**	**B ± SE**	**β**	***p*-Value**
Constant	−0.083 ± 0.046		0.094
HF without femoral neck BMD	0.011 ± 0.053	0.003	0.836
HF with femoral neck BMD	1.663 ± 0.387	0.893	**<0.001**
HF adjusted for lumbar BMD	0.327 ± 0.656	0.106	0.627
	R^2^ = 0.999		

## Data Availability

All the data are presented in this work.

## Abbreviations

The following abbreviations are used in this manuscript: BTM = bone turnover markers; BMD = bone mineral density; BMI = body mass index; DXA = Dual-Energy X-Ray Absorptiometry; group DM = type 2 diabetic population; group nonDM = non-diabetic population (controls); HF = 10-year probability of hip fracture; HF-FN = 10-year probability of hip fracture without femoral neck BMD (FRAX); HF+FN = 10-year probability of hip fracture with femoral neck BMD (FRAX); HF+T2D = 10-year probability of hip fracture with type 2 diabetes adjustment (FRAXplus); HF+LS = 10-year probability of hip fracture with lumbar BMD adjustment (FRAXplus); IQR = interquartile interval; MOF = 10-year probability of major osteoporotic fractures; MOF-FN = 10-year probability of major osteoporotic fractures without femoral neck BMD (FRAX); MOF+FN = 10-year probability of major osteoporotic fractures with femoral neck BMD (FRAX); MOF+T2D = 10-year probability of major osteoporotic fractures with type 2 diabetes adjustment (FRAXplus); MOF+LS = 10-year probability of major osteoporotic fractures with lumbar BMD adjustment (FRAXplus); N = number of patients; OGTT = oral glucose tolerance test; T2D=type 2 diabetes mellitus); TH-BMD = total hip BMD; YSM = years since menopause; vs. = versus).

BMD	bone mineral density
BMI	body mass index
BTM	bone turnover markers
DM	type 2 diabetes mellitus
DXA	Dual-Energy X-Ray Absorptiometry
FRAX	Fracture Risk Assessment Tool
HF	10-year probability of hip fracture
MOF	10-year probability of major osteoporotic fractures
N/A	not applicable
N	number of patients
OGTT	oral glucose tolerance test
PTH	parathormone
REMS	Radiofrequency Echographic Multi Spectometry
Q	quartile
SD	standard deviation
SE	standard error

## Appendix B

**Table A1:** Related samples test for equality of MOF, respective HF distribution within in non-diabetic subjects (BMD = bone mineral density, MOF = 10-year fracture risk of major osteoporotic fracture, Q = quartile).

10-Year Probability of Fracture (%)	Value
MOF without femoral neck BMD, median (Q1, Q3)	3.80 (2.70, 5.50)
MOF with femoral neck BMD, median (Q1, Q3)	3.90 (2.90, 5.63)
MOF adjusted for lumbar BMD, median (Q1, Q3)	3.00 (2.30, 4.30)
HF without femoral neck BMD, median (Q1, Q3)	0.60 (0.40, 1.70)
HF with femoral neck BMD, median (Q1, Q3)	0.59 (0.30, 1.53)
HF adjusted for lumbar BMD, median (Q1, Q3)	0.40 (0.20, 1.10)
